# An online survey of postmenopausal women to determine their attitudes and knowledge of the menopause

**DOI:** 10.1177/20533691231166543

**Published:** 2023-03-29

**Authors:** Rawan Aljumah, Samantha Phillips, Joyce C Harper

**Affiliations:** EGA Institute for Women’s Health, 4919 University College London, London, UK

**Keywords:** Perimenopause, menopause, postmenopause, hormone replacement therapy, Estrogen

## Abstract

**Objective:**

To explore postmenopausal women’s attitudes and knowledge of the menopause.

**Study design:**

An online survey to evaluate women’s attitudes and knowledge of the menopause, promoted via social media. In this study, only the data from 829 women who identified as postmenopausal were analysed.

**Main outcome measures:**

Quantitative and qualitative data.

**Results:**

Regarding women’s attitudes towards the menopause before they went through it, 18.0% were accepting of it, 15.8% were dreading it and 5.1% were looking forward to it. 38.1% of women felt that the menopause was difficult, 24.6% felt it was very difficult and 20.7% felt it was fine. 94.1% of women had never been taught about the menopause at school, and 49.0% did not feel informed at all about the menopause. More than 60% started looking for information regarding menopause as their symptoms started. The qualitative thematic analysis of the participants’ responses produced six themes: the need for education, knowledge and understanding of symptoms, why is getting treatment so difficult, feelings and attitudes towards the menopause, the impact of menopause on a woman’s life, the importance of the media – are they getting it right.

**Conclusion:**

Women’s lack of education and their healthcare professionals’ lack of adequate training on the menopause means that women enter this critical life stage uneducated and unsupported. It is vital that everyone is taught about the menopause and that general practitioners receive proper training. The negative narrative of menopause needs to be re-addressed to normalise the menopause and give postmenopause women hope.

## Introduction

The World Health Organisation (WHO) defines menopause as ‘the permanent cessation of menstruation resulting from loss of ovarian follicular activity’. In natural menopause, this occurs after 12 consecutive months of amenorrhoea.^1^ The proportion of perimenopausal and postmenopausal women is on the rise.^2^ By 2030, it is estimated that over one billion women across the globe will be perimenopausal or postmenopausal, and nearly 50 million women will be expected to reach the menopause each year.^3,4^ Since the median age at death of women in the UK is 85.8 years,^5^ the majority of women are expected to live a significant proportion of their life in the postmenopausal phase.

The health of perimenopausal and postmenopausal women should be a primary concern worldwide. Up until recently there has been no teaching provided on menopause in UK schools and minimal education for health care professionals (HCP). Health care professionals should be equipped with the knowledge and skills to help guide women through this critical life transition. Women should be provided with the information, appropriate assessment and treatments that will improve their quality of life and aid in health promotion into the post-reproductive years.

Lack of knowledge on the menopause negatively impacts women’s perceptions and attitudes towards the menopause. Our study analysing 947 perimenopausal women demonstrated that 90% of women had never been taught about the menopause at school, and over 60% did not feel informed at all about the menopause.^6^ When we asked women under 40 years of age, they were slightly more informed but about 80% had never been taught about the menopause at school and under 50% did not feel informed at all.^7^ The European Menopause Survey, which included participants from seven European countries including the UK, showed that women obtained information mainly from non-medical sources such as magazines and television which may not always reflect the correct information. It also demonstrated that women were indeed knowledgeable about the menopause since awareness of hormone therapy as a treatment option was present but their knowledge was not always correct and complete.^8^ The Asian Menopause Survey showed that 21% of women obtained information from friends, 13% from physicians and lastly from print and electronic media.^9^ It is vital to acknowledge that lack of education about menopause may leave women with their menopausal symptoms misdiagnosed or untreated; it may also expose some women to detrimental health effects.

Perception of menopause as an illness or as a natural biological process influences attitudes towards the menopause. Menopause-related symptoms have been found to be less prevalent in countries where menopause is viewed as a normal ageing process rather than a disease. For example, western and non-western societies have different attitudes towards menopause which influences how women experience the menopause.^10^ The European Menopause Survey revealed that the varied cultural expectations regarding menopause led to great variations between countries.^8^ Women in the UK had more severe symptoms and poorer quality of life compared to postmenopausal women in Spain and France. In contrast, the Asian Menopause Study revealed that women may leave menopause-related symptoms untreated due to the belief that menopause is a natural life process and are likely to choose alternative methods for symptom relief such as natural remedies or herbal medicines due to lack of knowledge of the treatment options available.^9^ It has been shown that women with more negative attitudes towards the menopause in general report more symptoms during the perimenopause.^11,12^

This study was part of an online survey done by Harper et al. exploring attitudes and knowledge of women over 40 years about the menopause and the perimenopause data has been reported.^6^ The aim of this paper was to explore postmenopausal women’s attitudes and feelings towards the menopause and to gain an insight into their lived experiences with the aim of helping develop educational resources.

## Methods

### Ethics

This study had ethical approval from UCL research Ethics Committee ID no: 9831/005. The selected sample population for the study were English speaking women who were aged 40 years and over at the time of entering the survey.

### Research design

This was a mixed-method, observational study. The online survey contained 34 quantitative questions and one qualitative question, using Qualtrics XM®. The survey was configured in a way that would allow separation of participants that considered themselves to be postmenopausal, perimenopausal and those that had not yet reached the menopause (other). Only respondents that identified as postmenopausal were included in this paper.

There were two sections to the survey.^6^ Socio-demographic information was required which included age; gender identity; sexual orientation; current relationship status; whether they have children and if so, how many, their highest educational qualification, field of work/study/trade, religion, ethnicity and disability status. The second section explored the participant’s individual experience, attitudes and their learning about perimenopause and menopause.

The survey was validated by conducting cognitive interviews with eight participants. Suggested changes were implemented, consequently, the menopause clinicians at UCH and some menopause experts including Magnificent Midlife, Behind the Woman and Know Your Menopause were approached for advice on further amendment. On completion of this process, a mini launch was run to check the survey’s functionality on Global Women Connected Facebook group (a group run by Professor Joyce Harper) and then the survey was promoted through various social media platforms of all authors including Twitter, Instagram, Linkedin and Facebook between 19 May 2021 and 26 May 2021. Data saturation was reached and evidenced during data analysis and confirmed during initial coding.

### Inclusion criteria

The inclusion criteria consisted of participants who were aged 40 years and above, were English speaking and identified as postmenopausal.

### Consent

Consent was given by the participants through a consent form in the first part of the survey.

### Data analysis

Quantitative data were analysed using descriptive statistics. The qualitative data was analysed by a thematic analysis method using a systematic six-phase approach suggested by Braun and Clarke. These six phases involve an iterative process of reading and re-reading the data with the aim of organising them into a collection of themes. Phase one involved reading the 594 responses of the participants that completed this question to familiarise with the data and recognise similar words and patterns that were salient throughout the responses. Phase two involved re-reading the responses while manually highlighting repeated patterns of responses for the purpose of forming initial codes. In phase three, the data was re-read and meaningful and repeated units of text were identified, which were assigned codes, allowing more data to be identified and categorised into these codes. Phase four involved reading the data again to a make sure that the text had been thoroughly read and analysed and the initial codes refined further so that they could be organised into themes; also, to ensure that no further codes emerged. Phase five was the process of describing, comparing and explaining the themes in order to clearly define and name them. Phase six was the writing up of the report where stages 1–5 have evolved to enable presenting the data in a way that delivers the overall essence of the data to the reader.

## Results

### Socio-demographic characteristics of the postmenopausal participants

A total of 829 women described themselves as postmenopausal and were included in this analysis (Table 1). The majority of the women in this study identified as female (810/829, 97.7%) and were married or in a civil partnership (530/829, 63.9%).

**Table 1.** Demographic characteristics of the postmenopausal women of the study.

	*n*	Frequency (%)
Age		
40–45	13	1.6
46–51	120	14.5
52–55	249	30.0
>56	447	53.9
Country of residence		
UK	718	86.6
Other	111	13.4
Gender identity		
Female	810	97.7
Non-binary	1	0.1
Prefer not to say	8	1.0
Other	10	1.2
Sexual orientation		
Heterosexual	762	91.9
Homosexual	27	3.3
Bisexual	19	2.3
Pansexual	1	0.1
Asexual	4	0.5
Prefer not to say	16	1.9
Relationship status		
Single	136	16.4
In a relationship not cohabiting	26	3.1
In a relationship cohabiting	93	11.2
Married/civil partnership	530	63.9
Prefer not to say	10	1.2
Other	10	1.2
Widowed	24	2.9
Parity		
1	158	19.1
2	307	37.0
3	103	12.4
4 or more	39	4.7
I do not have children	218	26.3
Prefer not to say	4	0.5
Highest educational qualification		
Secondary school	62	7.5
A level/college level	133	16.0
University undergraduate	164	19.8
University postgraduate	421	50.8
Other	44	5.3
Prefer not to say	5	0.6
Ethnicity		
White-English/Welsh/Scottish/Northern Irish/British	685	84.2
White-Irish	30	3.7
Any other white background	69	8.5
Black/Black British – Caribbean	3	0.4
Black/Black British – African	5	0.6
Any other Black/African/Caribbean background	1	0.1
Asian/Asian British – Indian	4	0.5
Asian/Asian British – Pakistani	0	0.0
Any other Asian background	3	0.4
Arab	0	0.0
Latino	1	0.1
Mixed ethnic background	7	0.9
Any other ethnic group	3	0.4
Prefer not to say	3	0.4

### Women’s attitudes and experience of the menopause

**Figure 1.**
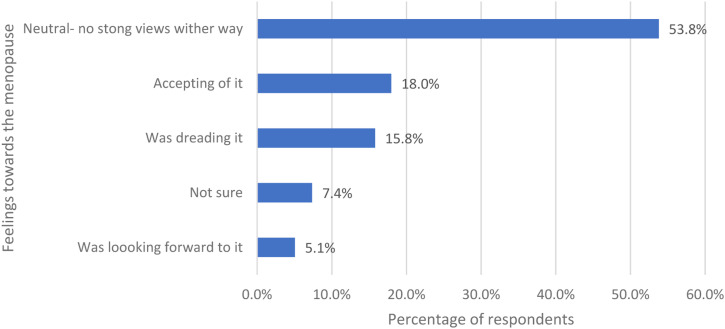
The women’s feeling towards the menopause before they went through it.

Women were asked how they felt about the menopause before they went through the menopause and were given five options (Figure 1). More than 50% (446/829) felt neutral (no strong views either way).

**Figure 2.**
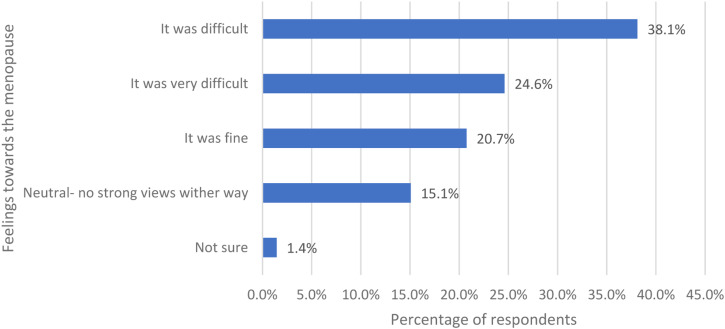
The women’s feeling towards the menopause now that they have been through it.

Women were asked how they felt about the menopause now they have been through the menopause and were given five options (Figure 2). Most respondents felt it was difficult (316/829, 38.1%) and 24.6% felt it was very difficult (204/829).

**Figure 3.**
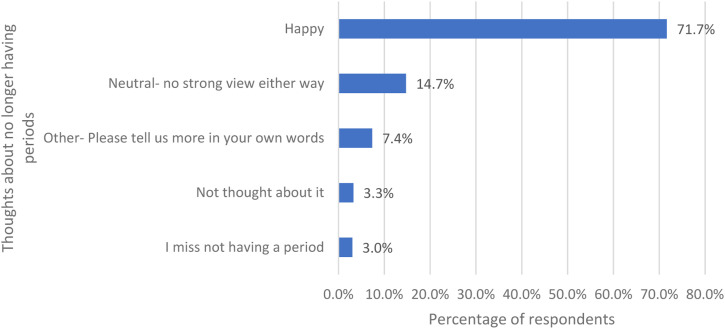
Women’s thoughts about no longer having periods.

Women were asked about their thoughts about no longer having periods and were given five options (Figure 3). Over 70% are happy about no longer having periods (594/829) and only 3.0% of respondents miss not having a period (25/829).

**Figure 4.**
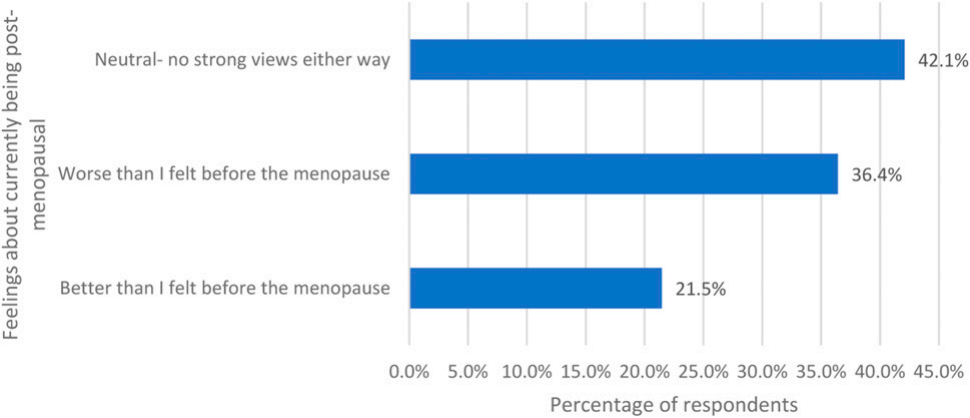
The women’s feelings now that they are postmenopausal.

Women were asked how they felt now that they are postmenopausal and were given three options (Figure 4). 42.1% (349/829) said neutral, 36.4% (302/829) said worse than I felt before the menopause and 21.5% (178/829) said better than I felt before the menopause.

### Menopause knowledge and education

**Figure 5.**
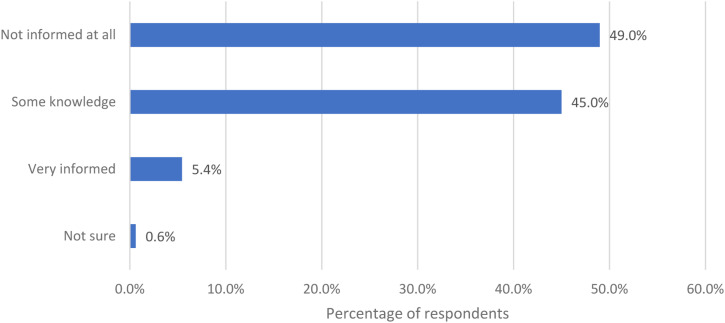
How informed the women felt about perimenopause/menopause before the age of 40.

Women were asked how informed they felt about the perimenopause/menopause before the age of 40 (Figure 5). Nearly half (49.0%) felt not informed at all (406/829).

**Figure 6.**
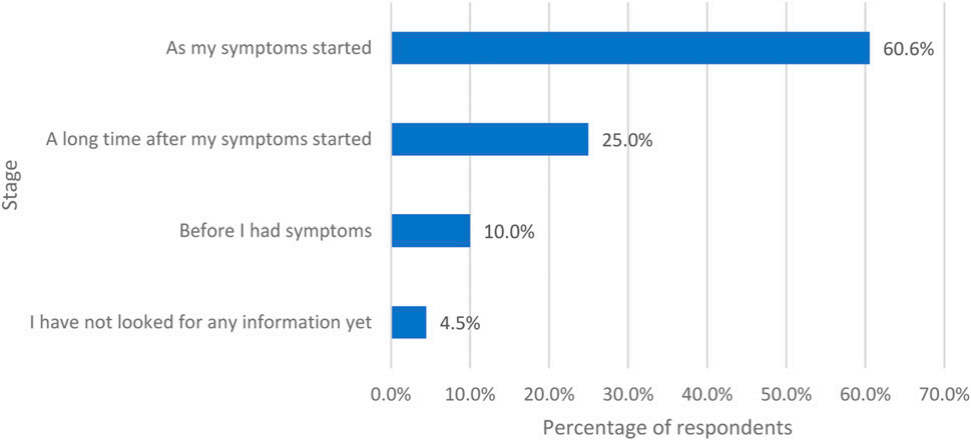
The stage at which the women started to look for information regarding perimenopause/menopause if they did look for information.

Women were asked if they had looked for information about the perimenopause/menopause, at what stage did they look for it (Figure 6). Most respondents looked for information as their symptoms started (502/829, 60.6%).

**Figure 7.**
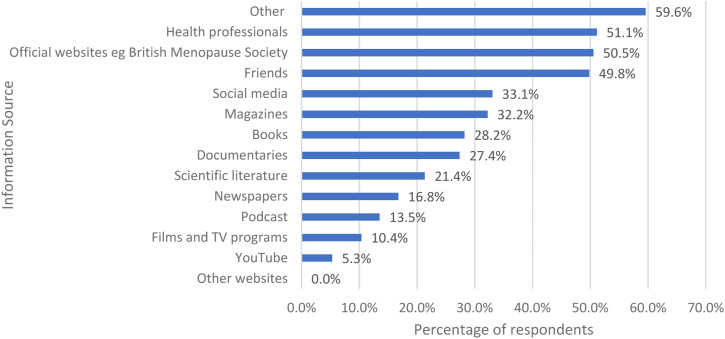
The ways in which the women looked for information about the menopause.

Women were asked if they had specifically looked for information about the menopause in any of these ways and they could tick all that applied (Figure 7). The three top answers were other (494/829, 59.6%), health professionals (424/829, 51.1%) and official websites of professional societies, such as the British Menopause Society (419/829, 50.5%).

**Figure 8.**
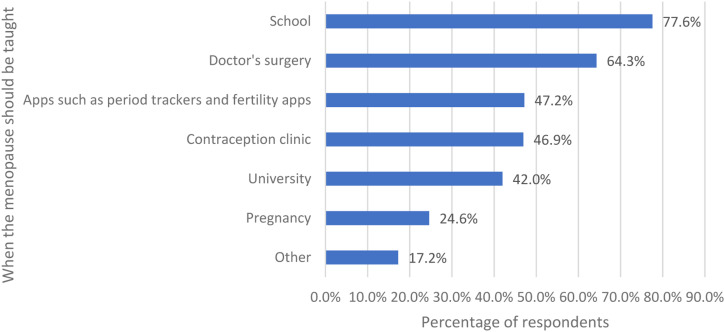
When the women thought that menopause should be taught.

Women were asked when they thought the menopause should be taught and were given a list of options (Figure 8). Most respondents wanted the menopause to be taught at school (643/829, 77.6%), doctors’ surgery (533/829, 64.3%) and through apps (391/829, 47.2%).

Women were asked if they had been taught about the menopause at school and were given three options. 94.1% (780/829) said not at all, 5.5% (46/829) said some basic information and 0.2% (2/829) had been taught very detailed information.

### Qualitative analysis

Women were asked if they would like to tell us anything about their views of the perimenopause/menopause in their own words. A total of 594 women gave answers. Six themes were identified: The need for education, knowledge and understanding of symptoms, why is getting treatment so difficult, feelings and attitudes towards the menopause, the impact of menopause on a woman’s life, the importance of the media –are they getting it right.

### The need for education

This was a major theme that many women spoke about. There were three subthemes identified regarding the need for education: their lack of education; their GPs/HCPs lack of education and education in schools.

#### Their lack of education

The women were shocked about how little they knew about the menopause. Some of the women have never even heard about perimenopause.

I found out I was going through the menopause when I booked a well-womens checkup with my doctor. My periods had been erratic, so I figured I might be starting to go through the change, then my blood test confirmed it. Also my periods have stopped for more than a year now so I’ve just learned from your questionnaire I’m post menopausal! And I’d never heard of perimenopausal till I heard a comedian use this word on tv just after Xmas this year. #758

I’d never heard of peri-menopause until the last few years. #541

Many women expressed how they did not realise that menopause was more than just hot flushes, as that was the only symptom they were aware of; also, they were shocked about what a significant impact the menopause could have on their life.

No idea of the many symptoms. I though night sweats & hot flushes we’re the only symptoms. I had neither. I was always hot. Thin quilt all year round. Vaginal atrophy was very bad until vagifem. Damage done though. Intercourse is not possible. No libido is hard. All the emotional aspects are hard. I would love to be the affectionate person I was. #815

Many women said they were not able to identify that the symptoms they were experiencing were related to menopause because of their lack of knowledge about the menopause. They also shared how lack of knowledge has had a negative impact on their health as they were not able to link certain symptoms to the menopause and it was never brought up by their GPs, leaving their symptoms inadequately managed. As a result of this, they did not know when to ask for help or if they needed help at all. They expressed how they felt unprepared for the changes they experienced and had no idea what to expect.

Wish I’d known more about it before symptoms, I reflect and now recognise I was peri menopausal in my early 40s - if only I’d been informed to recognise it. #819

Some of the women shared how ill-informed they felt about the menopause although they were healthcare workers and have post-graduate level of education, highlighting the extent to which the women lacked knowledge about the menopause.

I was shocked at how little I knew about the menopause despite being a healthcare professional myself… #482

The women wondered whether having knowledge regarding signs and complications of menopause could have helped them better cope and may have improved their quality of life during this period. One respondent shared that having information from relatives who worked in obstetrics and gynaecology made her confident with coping with symptoms throughout her menopause experience.

My husband and my son are obstetrician and gynecologist hence I had full information regarding menopause and had full confidence in overcoming it. #706

Several of the women shared similar thoughts about how, if they had been equipped with more knowledge, they would have been more prepared and had a more positive experience.

…If I’d been more informed about the menopause I might perhaps have worried less about the memory loss and brain fog and put it down to menopause rather than convincing myself I might be getting dementia! #38

One respondent shared how it was difficult to differentiate between menopause-related symptoms and symptoms that are situational due to different life events. For example, one respondent shared that her menopausal symptoms coincided with a bereavement, making it difficult to ascertain whether certain feelings were related to menopause or grief.

I was going through a traumatic time personally (bereavement) as I went through the menopause. It was hard to tell what was menopause and what was grief… #23

#### Their GP/HCP’s lack of education

The women were shocked at their GP/HCP’s lack of knowledge about the menopause. A few women were aware that the menopause was not a compulsory part of their GP’s CPD. They felt that there should be compulsory education for GPs and a requirement for regular CPD on the topic. Some of the women were able to access specialist care in menopause clinics and could clearly see the difference in knowledge.

… the wave of multiple symptoms got too much and I went to my gp to ask for HRT. I was shocked at how little she knew, googling things in my appointment and looking up treatments in the BNF. She was very sympathetic however. Unfortunately she prescribed me too high a dose of a combined tablet HRT and within a week I was suffering extreme anxiety and mood changes that forced me to stop. At that point we decided together that my multiple symptoms were too complicated and she referred me to a specialist menopause clinic. When I was seen there the difference in knowledge was staggering and I was so relieved to be given the right HRT. The specialists told me how GP education was organised and I was shocked to hear that menopause is not a compulsory part of their CPD… #482

A lot of women expressed how they wished their GPs were better informed. Many of them would leave their GP consultations feeling like they were being dismissed and unsupported.

GP hasn’t been very helpful but think it’s because she isn’t knowledgeable on subject even though she’s a female of menopausal age. I feel like I have now got nowhere to go with menopausal issues such as no sex drive. #274

Several women shared examples of how they felt their GPs lacked knowledge about the menopause as they were not able to identify presenting symptoms as related to the menopause and sometimes even misdiagnosing them.

I went to the doctor when I had a flushed face/cheeks around 5 years ago. He diagnosed rosacea and gave me some topical gel. In hindsight I think it was probably the start of per-menopause but the doctor did not mention anything about menopause. The gel he gave me didn’t work but the flushed face symptom has now disappeared over time… #770

#### Education in schools

The women felt that menopause should be taught at school and that everyone should learn about menopause, including males. They expressed how teaching children at a young age about the menopause would normalise it and reduce the stigma menopause is associated with. One respondent used pregnancy as an example to demonstrate how teaching people about a certain aspect of women’s health makes it accepted and not a taboo subject.

Without a doubt, we need to be teaching children - from teenagers onwards - about the menopause and what it means physically and mentally for those going through it and its various stages. Only by it becoming as normalised as discussing pregnancy will both sexes fully appreciate the impact and effects of it on women (and their partners…) #871

This should be taught at school along with menstrual cycle in full detail. #531

Many women emphasised the importance of both genders being educated about the menopause so that they could have a better understanding of what goes on.

Should be a much more common topic to discuss with girls (and boys). It’s something that HUMANS will need to adjust to -- boys marry girls who grow to up have menopause. #258

### Knowledge and understanding of symptoms

The poor knowledge and understanding of symptoms related to the menopause led to five subthemes: surprised by length of symptoms, heavy periods, mental health impact, cognitive impact and loss of identity.

#### Surprised by length of symptoms

Many women shared their disbelief that they were still experiencing symptoms related to menopause. Some women even expressed how they sometimes felt worried that their symptoms would never go away. Some of the women had a preconceived idea that menopause was something that you just went through and came out the other side from.

Does it ever get better. I started with night sweats and hot flushes aged 42. I am now 66 and they are still awful and debilitating. #318

I never expected it to be so awful. Ten years since I became postmenopausal I am still experiencing psychomotor symptoms. #297

#### Heavy periods

The heavy bleeding the women experienced during the perimenopause had left its mark as they had struggled with it before becoming postmenopausal. Some women even went as far as saying that the heavy bleeding was the most difficult part of menopause and one woman explained her periods became so heavy that she required intravenous fluids.

…After 24 months with no period, 6 months ago I had a ‘final’ heavy period and then a UTI… #411

Did not know what it was. Had horrendous periods for about a year around the age of 51. Heavy bleeding. Very irregular. Went to GP given iron tablets and sent away told probably start of menopause… #321

#### Mental health impact

Many women shared the mental health struggles they experienced as a result of their menopause experience. Some even expressing that the mental symptoms were far worse than the physical symptoms. Some reached the point of suicide or were having suicidal thoughts.

…I think many people are only aware of the physical symptoms but for me the psychological/mental health issues were/are far harder to deal with. #153

Nightmare. I was suicidal at times. I was a danger to myself and other people. #590

Many women also expressed how depressing their experience was. Some of the terms the women used to describe their mental state included sad, depressed, a total mess and low. A lot of the women were shocked by the unexpectedness and significant impact the menopause has had on their mental well-being.

…It is debilitating especially the deterioration in mental health which I was not expecting. I had anxiety, depression & paranoia were crippling. I had suicidal ideation and it is only because my partner lost his 53 year old sister in law to suicide that I didn’t go through with it because I couldn’t put him through that much pain again. However, it was a very close call at times… #522

The women explained how the lack of knowledge contributed to their anxiety due to the uncertainty of whether their symptoms were menopause-related, how long they would last, how they could manage them and if any further symptoms are to emerge. Some of the women are still suffering from anxiety.

I had expected hot flushes as that is what women seem to concentrate conversations on but I hadn’t expected the aches and pains, anxiety, panic attacks, loss of confidence and other symptoms. I had been on HRT and was forced by my GP to come off it aged 59 I suffered the symptoms for 4 years before asking to be put back on to HRT and was given the lowest dose. I’m now 67 and dreading the day when I’m made to stop taking HRT again. Some women never come out the other side and have to suffer for the rest of their lives. #74

#### Cognitive impact

Many women emphasised how difficult and challenging the cognitive symptoms were, such as difficulties with concentration and recall. Women were saying how failing to remember things was affecting their day to day lives such as their ability to work to their potential professionally, which has in turn impacted their confidence. The cognitive symptoms have also had impact on the quality of life of some women affecting their sleep quality.

I felt very anxious I felt I was doing My job wrong. Could not remember the things I know normally… #715

The cognitive function was the worse, I was in a senior Director role and just couldn’t function. Also worry my memory is so bad that I have dementia. #696

#### Loss of identity

Several women shared that they did not feel like themselves and felt a loss of identity and for some of the women, these feelings are still ongoing.

…Now I’m on the other side, I’m back to being myself except I have feelings of regret for what feel like several lost years while I was not myself. #322

… I still have times when I’m struggling with the after effects and I’m not the same person as before. Having no hormones seriously fucks you up #462

Many of them shared how they felt less confident. Some of the women described the physical changes to their appearance that resulted from the menopause such as a reduction in muscle mass, vaginal atrophy, growth of facial hair and weight gain.

My only issues relate to having no libido, vaginal dryness, and obvious changes with my skin (skin is no longer “plump” and looks older — also growth of a few facial hairs I don’t want!) … #209

The women explained that these changes had an impact on their mental well-being and self-perception. One woman even shared how her body and mind felt like they did not belong her.

…My mind and body feel like strangers. It has been awful. #237

### Why is getting treatment so difficult?

The difficulty some of the women faced in getting adequate treatment and care was a major theme that many of the women shared in their responses, especially within three subthemes: specialist care, HRT and early menopause.

#### Specialist care

Several of the women shared how they wished there was more specialised care available to them and suggested having a menopause specialist in their GP surgeries.

Women need better access to menopause clinics & to be encouraged not to feel they have to ‘put up’ with debilitating side effects which impact the quality of life. So many women I know had to fight for years to get a referral. Better information about the range of treatments available should be provided by GPs. GPs need better training… #417

Need at least one GP in a surgery to specialise in menopause. #261

The women in our study shared how they were provided with wrong or inappropriate treatments. A lot of the women spoke about different approaches to management such as making dietary alterations and resorting to complementary therapies. Several of the women shared their experiences about having other health conditions and how that has impacted the way they were able to manage the menopause in terms of what management options they were able to or are allowed to have.

…I found that when I radically altered my diet, to a whole grain, higher protein, high veg, sugar free (mostly) diet and took up dance I was able to manage my peri/menopause well…#898

I took HRT for 6 months, I was then diagnosed with breast cancer 7 weeks ago and can no longer take HRT due to the type of breast cancer I have… #393

Many of the women shared stories of how they felt dismissed by their doctors with some even pointing out the gender of their doctor almost like they expected female doctors to be more supportive and understanding, yet they still received unsatisfactory care.

Although I had a number of symptoms, they were mild so I didn’t really worry & when I mentioned it to my (female) Dr she wasn’t interested… #503

#### Hormone replacement therapy

Many of the women shared their frustration about not being offered HRT because HCPs failed to identify or even consider that symptoms patients were presenting with could be menopause-related.

…I really thinks my initial symptoms of early waking, anxiety, hot flushes could have been peri or menopausal and my anxiety just progressed into bad depression. Never suggested by any health professional it could be that and never offered HRT. I have osteoporosis and on alendronuc acid now so perhaps HRT could have helped with that too… #697

Some women never even approached their GPs until much later as they had no knowledge that the symptoms they were experiencing related to the menopause. Several women shared how difficult it was to get HRT prescriptions from their GPs; one respondent shared that her GP only prescribed her HRT because she experienced a hot flush during her consultation. Another woman shared that she suffers from osteoporosis and how this could have been prevented had her GP offered HRT to her. One respondent shared how she had to beg to have HRT prescribed to her and that she was only able to get a prescription when her GP realised that her husband was affected by her symptoms. On the other hand, the women who were offered HRT described how their HCPs failed to explain the advantages and risks associated with HRT.

Drs are not trained, drs are misogynistic and sexist (would not give me HRT unless husband approved and then only to improve husband’s quality of life, mine is of no importance). Help is extremely difficult to find, drs are not up to date on scientific literature, drs are hurting women. I will likely eventually commit suicide because of the lack of help and misinformation. I have had to spend enormous amounts of $$$ for private specialists (insurance doesn’t cover). The trauma I now have from uneducated, misogynistic Doctors makes me just avoid them altogether. There really is no help, even if you have the strength and education to stand up to them. You are utterly alone on this trip, society does not care. No one cares. No wonder suicide becomes the only option to end the misery. #121

Many of the women shared their hesitancy to commence HRT because of worries about its link to cancer. Some women shared their uncertainty about initiating HRT due to a family history of cancer. Several women shared how they wish that their HCPs had discussed with them alternatives to HRT.

…I was concerned about taking HRT as I thought there was a link between the increase of cancer if you had done fertility treatment and then took HRT. I couldn’t get an answer to this… #506

Some of the women shared how commencing HRT was helpful in alleviating some of the symptom burden. Some even went as far as saying that initiating HRT was the best decision they have ever made and even lifechanging. Some were even worried about when they would eventually have to stop HRT treatment.

HRT has been fabulous-really helped with symptoms and best of all helped me to sleep better than I ever have before. #78

#### Early menopause

Some women shared their experience with having an early menopause due to medical or surgical reasons. A lot of these women shared how it was challenging having to deal with the ‘suddenness’ of menopause and how they were not provided with adequate support afterwards, sometimes none at all. Many of these women also expressed there should be increased awareness of early menopause.

I had sudden-onset menopause after a total hysterectomy due to a large fibroid. This meant that all my symptoms appeared very suddenly and I wasn’t prepared at all for their severity. The doctors had told me that I would be menopausal straight after the operation but they didn’t explain what that could look like…. #883

It can be such an individual journey/arrival. With hindsight, I can see that I was happy with a gradual ‘slide’ through the perimenopause until a very large ovarian cyst was found and ultimately a surgical menopause sent me straight to post-menopause overnight. That made all the symptoms I experienced arrive suddenly and with great impact… #573

### Feelings and attitudes towards the menopause

In general, the women had mixed feelings towards the menopause including negative and positive feelings.

#### Negative feelings

Many women shared negative feelings towards the menopause and their menopause experience, and used various adjectives to describe this such as miserable, horrific, devastating, lifechanging, awful, debilitating etc. Many women explained how it was ‘the most horrific time of my life’. ‘Very difficult and challenging to live with’. ‘Quite simply the worst thing I have ever had to deal with’.

Some of the women shared how their experience felt very isolating which was related to the lack of support and the stigma surrounding menopause. Many of them did not feel comfortable enough to talk about what they were going through.

…Even though you know women are feeling exactly like you it’s still so very isolating… #927

Several of the women shared how they now felt ‘old’ and how they felt that the menopause signified ageing. They shared how the menopause meant that their reproductive years had ended and the feelings of sadness and loss that came along with that.

Had a big impact on my life. Hot flushes were ok but felt complete disconnect from my family. Wanted to go to work as this was the only area I enjoyed. Complete loss of sex drive which hasn’t returned, major impact on my personal life. Don’t know how to fix it. Suddenly feel much older #665

#### Positive feelings

For many women, the menopause brought a sense of relief, whether it was because it meant relief from having periods or relief from suffering they experienced throughout their reproductive years attributed to menstrual pain.

I was ecstatically happy when my periods stopped as I had severe PMS since I was 11 when my periods started… #216

My periods were horrible: unpredictable, heavy, long, painful, debilitating. I started late (age 15) and ended early (age 48), with few and/or mild symptoms of menopause — and I am ECSTATIC to be rid of periods… #60

Some women felt that this was a normal life stage and should be regarded as such. The women expressed how they felt a natural process was being medicalised and how women should just learn how to live with menopause-related changes and symptoms and find ways to cope with them.

… Night sweats were the worst, having to change bedding and nightwear a couple of times a night and still function as a normal human being! Having said that, it’s a normal part of the human beings' life cycle. I realise that we do and have stuffed our bodies full of chemicals… #499

### The impact of menopause on a woman’s life

The women shared a lot about the impact the menopause has had on various aspects of their lives including their personal and professional life. They shared how this was made worse by lack of support and lack of information available to them, with many of them expressing how they wished they had more opportunities to talk to others about what they were going through. This major theme led to five subthemes: information, talking to others, lack of support, menopause and the workplace, impact of menopause on relationships.

#### Information

Many women expressed how there is not enough information readily available about the menopause. Many of the women faced difficulty finding relevant, reliable resources. Some of the women had to do their own research about the menopause because information was not easily accessible. Others had to ask friends and family for information.

I wish I had been better informed of all the different symptoms. Why was it so hard to find any useful info. Why was my GP so unhelpful? #734

Hear others in work talking about hot flushes. That’s what I was expecting. Very busy working mum of two kids with disability. Hadn’t much time to dwell on it. For me the brain fog, problems with memory, night sweats and anxiety were the worst. Only after a conversation with a friend did I realise that these are all symptoms and then did some research. Not enough awareness or conversation about menopause and how debilitating it can be out there. #574

Several of the women shared how deeply impacted they felt by the amount of misinformation out there.

It was a challenging time with effects that came on gradually though the affects were devastating. I feel there is a lot of misinformation and it’s hard to get correct information #226

A lot of the women wished they had more knowledge earlier in their menopause experience to have been able to cope and address their concerns more promptly and not suffer for as long as they did. Many of them expressed how they felt that if they had been better informed, they might have been more prepared and had a more positive menopause experience. Several of the women called for more research about the menopause sharing how they felt that more should be done.

It’s just soooooooo underrepresented and misunderstood. Tons of research is needed, especially about the effects on emotions, psyche, self image etc. #436

#### Talking to others

Several of the women shared how being able to speak to other women who were going through a similar experience was very helpful; this made the women feel less alone in their experiences and acquire more knowledge about the menopause overall. For some, this involved online support groups and workshops; for others, it was talking to a friend or a colleague at work. Other women wished that there were more opportunities for women to be able to connect and share their experiences and some took it to themselves to join support groups.

I wish there was a chance to have menopause and peri menopause discussed in women’s groups before it happens and as it happens. #404

I had a hysterectomy due to endometriosis, no one in the medical profession warned me what/could happen with the lack of estrogen in my body. When I was experiencing the symptoms above, my GP put me on antidepressants. I have felt I was going crazy with my symptoms until I joined a Facebook group where there are thousands of women saying the same thing. #472

Some women were only able to link the symptoms they were experiencing to menopause after talking to others who shared their experience with them.

I’ve learned more about this subject through sharing experiences on a running group Facebook page for women of menopausal age. Didn’t know that so many of the symptoms I had experienced were connected to menopause. #685

Many women shared how there was still stigma surrounding menopause and felt they could not openly talk about the menopause freely. Women shared how they found talking about the menopause embarrassing. Many expressed strong feelings that the menopause should be normalised and that it should be talked about openly. Several women shared how the topic of menopause was never brought up with their mothers.

…My mother had never spoken to me about menopause and I found myself totally in the dark about it. My advice to friends and to my daughter is to talk about it and ‘normalise’ it - shake off the taboo nature it still has in society. #680

#### Lack of support

Many women shared how they felt their HCPs were generally unsupportive and unempathetic and that it was difficult for them to find other sources of support as they went through what could be quite a challenging life phase. Some women even shared how they had to see more than one GP before they felt that they finally got the support and care they were seeking and desperately needed.

At first I found it difficult to get a sympathetic hearing or help from my GP. The first GP I saw seemed very unsympathetic and told me this was a natural process I would just have to put up with. After two years of suffering I returned and saw a female GP who prescribed HRT. This changed my life. Unfortunately she retired soon after! #555

Many women shared their GPs were dismissive and unsupportive about their concerns and issues. Many of the women emphasised the importance of having more support for women going through the menopause.

…My doctors have never been very supportive. There’s just not enough support for women in the doctors surgery in my opinion. I hope this changes for my daughters future as she’s already experiencing similar symptoms to my fertile years. #927

Several of the women spoke about early menopause due to medical and surgical causes. They shared how they felt they were not supported following their transition to a sudden postmenopause; the doctors failed to explain what menopause would be like and what kind of help and support is available to patients or how to access it.

A few women expressed how they felt that women’s health is not taken as seriously as men and that if this was an issue affecting men more would be done and their concerns would be addressed with more urgency.

#### Menopause and the workplace

Many women spoke about the lack of support in the workplace and how negatively impacted they were as a result of this; this included losing their jobs.

It was a huge part of destroying my career, and yet no one said anything, or suggested that my violent mood swings, inability to concentrate, etc., were perhaps part of a physical issue rather than simply stress… #401

Several of the women felt that the lack of measures to support women’s experiences of menopausal symptoms and changes made it more difficult to cope. One woman shared how embarrassed she felt to sit in a male dominated meeting whilst experiencing hot flushes compared to a meeting where there were other women who were able to laugh about menopausal symptoms and make the situation less uncomfortable for everyone. They also reported issues with their periods.

For me the biggest shock, and most difficult issue was sudden, incredibly heavy periods. These could go from zero to a gush of blood in seconds — often they were so heavy an extra thick sanitary pad would flood almost immediately. At the time, I was working, in a senior and very challenging role and this had a huge impact on my life #449

Many of the women shared how difficult it was to cope with their symptoms and expressed their desire to have more awareness and support in the workplace. Employers are not very supportive or understanding.

…Employers and the workplace need to be educated and be made aware of the effects of the menopause on an employees' physical and mental health and the dehabilitating effect this can have. #171

#### Impact of menopause on relationships

Many women explained what a significant impact menopause had on their relationships and led to relationships deteriorating. The reasons for this were varied between women and included a reduced sex drive and lack of a supportive, understanding partner. For some women, menopause-related symptoms were so debilitating that despite having a supportive partner, menopausal symptoms caused tension in their relationship. Several women shared how they became more aggressive in their relationships with one respondent sharing how she would initiate arguments with no regards to what outcomes her actions would have.

…I was ridiculously short tempered and sometimes violent - I slapped my children… #880

My experience of going through the menopause was awful. It was really tough, not only for me but for my very patient and understanding husband. At one point I wouldn’t have blamed him if he left me - I was a total mess. I used to say that my next mood swing would be in 5 seconds and sometimes I thought that I was losing my mind. #550

### The importance of the media – are they getting it right?

The media was mentioned by several of the women and the effect the media has on menopause awareness and perceptions. Some of the women were not aware of menopause/perimenopause or issues related to them until they had heard about them through the media.

…I feel like I escaped lightly with my symptoms (compared to my mum!) but I now have new worries (that I did not have until I watched the programme on TV with Davina!) - I had not considered what my body 'loses' during the menopause and how I might be affected afterwards (e.g., brittle bones). Now I am considering taking vitamins or similar supplements… #38

Some women shared how the media helped them gain a better understanding of the menopause, and to recognise management options.

… I sailed through, perhaps because I took the supplements, which I learnt about after watching American TV programs by an OBGYN doctor who advised taking phytoestrogens from 10 years prior to menopause, and I started taking these at age 46… #647

Some of the women shared their frustration at the media’s negative portrayal of what menopause looks like and what it represents. The women shared how this reinforces focus on negative aspects related to the menopause and impacts menopause experiences. Several women shared that a more positive portrayal of the menopause would contribute to a more positive experience for women undergoing menopausal changes and even empower them.

As a middle-aged woman I find media coverage that catastrophises the menopause very undermining. The implication that every woman over 45 will soon become an unreasonable bitch and an emotional, physical and cognitive wreck due to the menopause is really damaging, especially professionally. Most of us do just fine. If anyone has medical problems with the menopause they can take it up with their doctor just like any other medical condition that men and women may suffer at some point in their lives. Let’s have more positive coverage of the menopause, and greater awareness that for most women it is either a minor inconvenience or a non-event… #556

Other women shared their delight that the media is now highlighting such an important topic which leads to increased awareness about the menopause.

Definitely more menopause education needed for everyone. TV and radio documentaries are often a good initial introduction. Newspaper and magazine articles too. #14

## Discussion

The purpose of this study was to hear what postmenopausal women felt towards the menopause so that we can improve women’s health education.

### Women’s education

It was clear that many women in this study were uninformed about the menopause and had not been taught about it at school, which was similar to what we found in perimenopausal women^6^ and women under 40.^7^ An Italian study analysing women aged 45–60 years found that more than half of the participants did not receive any information about menopause and possible therapies, and those who did, often rated it as poor and contrasting.^13^

Research has demonstrated the direct correlation between the severity of menopausal symptoms and a woman’s level of knowledge.^14^ Women who have a higher level of education are better informed about menopause symptoms and are more aware of these symptoms and methods of coping with them; they are also more likely to seek treatment for their symptoms. We are optimistic that this situation will be improved in the UK now that menopause education has been added to schools’ RSE curriculum.^15^ In our survey of women under 40, we found slightly more knowledge than women over 40 (7) but we need to be at a point where all women feel very informed.

The qualitative analysis revealed the women’s desire to have a space to connect with others going through the same experience and to share their knowledge; they brought up online support groups and menopause workshops as examples. A study investigating the effect of education via support groups on early symptoms of menopause showed that participants in the support group had a reduced frequency of hot flushes compared to those in the control group 4 weeks after intervention, showing a support group as a suitable educational method to enhance women’s health and increase their coping with menopause symptoms.^16^

The qualitative analysis also illustrated the women’s demand that men and boys are educated as well about the menopause and its impact. A Husband’s education affects the quality of life of postmenopausal women, especially in the psychosocial dimension, which may be due to a better understanding and supporting were their wives.^17^

### GP’s education

Our survey showed that over half of the women had looked for information about the menopause from health professionals. A study by Utian et al. found that more than a third of the women surveyed reported getting most of their information about menopause and women’s health issues from a physician.^18^

However, the women were angry and frustrated by the lack of knowledge their GPs had. Their experiences with their GPs were varied from being dismissed about their symptoms, receiving misinformation or no information at all, receiving inappropriate treatment and overall feeling unsupported and lacking confidence in their GP’s knowledge. The minority of the women who did receive adequate, satisfactory care from their GPs highlighted the culture of poor healthcare for women by attributing their positive experiences to luck.

A 2022 study exploring the views of GPs on their levels of confidence and comfort when advising or treating menopausal women demonstrated that GPs require better medical training to help them advise and treat women with menopausal symptoms.^19^ The majority of women in our survey expressed the need for improved training on menopause during medical school and GP training. Similar results were found in a study assessing menopause education of American obstetrics and gynaecology residents where only 20.8% of the 510 respondents reported that their program had a formal menopause learning curriculum.^20^

The qualitative analyses revealed some of the women’s concerns were that their GPs are using outdated information to inform decisions regarding treatment plans, with some women being refused HRT prescriptions by their GPs. Some studies have indicated that the flawed findings of the 2002 WHI study are still being used by some HCPs as the basis for their advice to patients even though there have been more current studies and reanalysis of existing data showing contrasting results.^21^ Access to HCPs who are up to date with evidence-based menopause care is key to make sure that these women receive the optimal care that they deserve.

### Women’s attitudes towards the menopause

Women in our survey had mixed responses regarding their attitudes towards the menopause ranging from the perception that the menopause is a normal life phase or transition, positive feelings in the form of relief from pain or the burden of the management of menstruation, and negative attitudes related to concerns or personal experiences of mental instability, signs of ageing and loss of fertility. Menopause is a natural life event and certainly in the UK, is in danger of becoming overmedicalised and being defined as a disease which needs treatment.^12^

The majority of the women in our survey, when asked how they felt about no longer having periods, felt happy. When asked how they felt about their feeling towards the menopause before they went through it, 53.8% said they had no strong views and the second most common answer was that they were accepting of it (18.0%). In our perimenopause study, 38.7% of women were accepting of the perimenopause/menopause and 31.0% were dreading it, which was the second most common response.^6^ Cheng et al. stated that while women with negative attitudes were younger and premenopausal, postmenopausal women tended to have more positive attitudes towards menopause.^22^ This is corroborated by a study that found that postmenopausal and older women consistently expressed more positive feelings about menopause than younger women in their forties and younger.^23^

The media plays a significant role in shaping perceptions and public opinion and feeding into the taboos and myths around menopause. Interestingly, many of the women in our survey resorted to the media to look for information about the menopause which included social media, podcasts, magazines, film and TV programs and documentaries. Unfortunately, the media might sometimes place an emphasis on the negative aspects of perimenopause, rarely emphasising any positive aspects, which may increase women’s anxiety and apprehension about the menopause. Changing the narrative by normalising menopause and emphasising positive or neutral aspects of postmenopause, such as freedom from menstruation, pregnancy and contraception, together with information about managing troublesome symptoms, might empower women to manage menopause with greater confidence.

### Impact of the menopause

The findings in this study have demonstrated the magnitude of menopause’s impact on all aspects of women’s lives. The majority of the women, when asked now that they have been through the menopause how they felt about it, said it was difficult (38.1%) or very difficult (24.6%). Similar findings are observed in a study revealing that 45% of women in the UK experiencing menopausal symptoms found them distressing and around 10% as severe.^24,25^

Many of the women in our study experienced a loss of identity and felt like their body did not belong to them as a result of these changes. Similar findings regarding apprehension towards weight gain, bodily changes, vaginal symptoms and ‘inhabiting a changed body’ have been found by a systematic review of qualitative evidence of women’s experience of the menopause.^26^

The qualitative analysis revealed the impact menopause has beyond physical health. Women who suffer menopausal symptoms such as hot flushes, night sweats and vaginal dryness are more likely to report anxiety and depressive symptoms^27^ 47% of the women in the Nuffield Health Survey reported feeling depressed and 37% suffered from anxiety.^28^ It is important to note that mid-life is a time of transition and stressful life events from divorce to a second career, combined with the physical symptoms of the menopause, can result in feeling overwhelmed.

The menopause has also had a significant impact on the relationships of the women in our study causing strain between couples and leading to relationship breakdowns. Other studies have had similar findings such as the survey by Currey et al. which revealed that the menopause had impacted around half of the respondents’ home life; 23% of the women reported feeling isolated from their family, 15% feeling they were a burden and 11% feeling that they were a better parent before the menopause.^29^ In the CLOSER survey, 39% of men reported that impact of menopause on their intimate relationships was worse than they anticipated.^30^ The MATE survey also demonstrated the impact menopausal symptoms can have on a woman’s partner with 63% of the men in the survey reporting they had been affected by menopausal symptoms. Specifically, the men noted that the symptoms put an emotional strain on their relationships, impacted intimacy and contributed to sleeping difficulties.^31^

Our study revealed the magnitude of the impact of menopause within the workplace. These findings are corroborated by a recent survey conducted by the Chartered Institute of Personnel and Development (CIPD) reporting that 59% of working women aged 45–55 years are negatively impacted by the symptoms they are experiencing within the workplace.^32^ The CIPD survey also revealed that 30% of women had to take a sick leave as a result of their symptoms.

Women make up almost 70% of the local government workforce and almost three quarters the workforce are aged between 40 and 64 years, which means that a significant proportion of employers will be experiencing menopausal symptoms at any given time.^33^ It is crucial that employers offer awareness and support female employees and are understanding of their experiences. Methods to implement this is to provide internal organisational guidance, ensuring there are policies that support women going through the menopause, encouraging women to seek help when necessary and ensuring there are clear pathways present for how to access such help, and training employers about the menopause.^34^

### Limitations of the study

All surveys are limited and biased by the people who choose to complete them.^6^ It is common for surveys to be completed by highly educated people, of white/British ethnicity, which compromises diversity. To overcome this, we currently have the same survey live, specifically aimed at Black women. And we have completed a study on the views of women under age 40 (7). It may also be the case that those with a negative menopause experience are more likely to complete a survey and this must be taken into account. In particular related to experiences with healthcare professionals, many women receive excellent care from primary care, and may be less likely to complete a survey. Another limitation is that the survey was only promoted on social media, so only women who have access to social media could participate.

## Conclusion

This study is important as it provides insight into the lived experiences of postmenopausal women, which is a scarce area of research. The data shows that women have a lack of education about this key life stage. Together with a reported lack of education from their healthcare professionals, women may be left undiagnosed and unsupported. We need to ensure that all health professionals have menopause training so they can give women information on managing their symptoms and well-being and this has been addressed in recent years, such as the courses led by the British Menopause Society. Women with severe symptoms may need to be referred to menopause experts. But we must not overmedicalize the menopause and present a totally negative narrative. We should give women hope that life postmenopause can be a fruitful and exciting time of their life.

## Supplemental Material

Supplemental Material - An online survey of postmenopausal women to determine their attitudes and knowledge of the menopause Click here for additional data file. Supplemental Material for An online survey of postmenopausal women to determine their attitudes and knowledge of the menopause by Rawan Aljumah, Samantha Phillips and Joyce C Harper in Post Reproductive Health
