# Supplementary material for: An online survey of postmenopausal women to determine their attitudes and knowledge of the menopause
Source: Post Reprod Health. 2023 Mar 29;29(2):67–84. doi: 10.1177/20533691231166543 (PMC10273865; doi:10.1177/20533691231166543)
Supplement: Supplemental Material - An online survey of postmenopausal women to determine their attitudes and knowledge of the menopause [file sj-pdf-1-min-10.1177_20533691231166543.pdf]

Supplementary 1

The Survey

Evaluation of how women learn about the perimenopause and menopause and their attitudes

UCL ethics committee approval
9831/005
Title of Study
Evaluation of how women learn about the menopause and their attitudes to the menopause.
Department
UCL EGA Institute for Women's Health.
Name and Contact Details of the Principal Researcher
Professor Joyce Harper. Institute for Women's Health, University College London.
Email: joyce.harper@ucl.ac.uk Telephone: 07880 795791.
Researcher: MSc student Samantha Phillips.

1. Introduction.
You have been invited to take part in a University College London, Institute for Women's Health research study being conducted to evaluate how women learn about the perimenopause and menopause. Before you decide it is important for you to understand why the research is being done and what participation will involve. Please take time to read the following information carefully and discuss it with others if you wish. Ask us if there is anything that is not clear or if you would like more information. Take time to decide whether or not you wish to take part. Thank you for reading this.

2. What is the project's purpose?
All women will go through the menopause but menopause education is almost non-existent. As a result, very few women know about the common symptoms of the menopause and many will suffer peri/menopausal symptoms for a number of years without realizing the cause. In this survey we aim to understand more about when and how women are learning about the peri/menopause with the aim of improving peri/menopause education. As is routine when doing surveys, we will ask you some questions about your demographics, such as your religion, ethnicity, sexual orientation, and age so we can determine the type of women who have completed the survey.

3. Do I have to take part?
It is up to you to decide whether or not to take part. If you do decide to take part you will be asked to answer the questions in the online survey. You can withdraw at any

time without giving a reason – simply do not submit your answers. If you decide to withdraw before completing the survey, your answers will not be used in the study. Once you have submitted your answers, we cannot withdraw them as the survey is anonymous.

4. What are the possible disadvantages and risks of taking part?
The UCL Research Ethics Committee have approved this study, and we do not anticipate any risks to any individuals taking part in this study. After completing the survey, we will direct you to the menopause poster which has additional information. If you have any concerns about the answers to the questions in this survey relating to your health, you may wish to contact your doctor.

5. What are the possible benefits of taking part?
You may learn some information about the menopause.

6. What if something goes wrong? I
If you have any complaints regarding your treatment by the researchers, you can complain to Professor Harper on joyce.harper@ucl.ac.uk or to the Chair of UCL Ethics. In the unlikely event of something serious occurring during or following your participation in the project, please also contact Professor Harper. However should you feel your complaint has not been handled to your satisfaction please contact the Chair of the UCL Research Ethics Committee – ethics@ucl.ac.uk.

7. Will my taking part in this project be kept confidential?
All the information that we collect about you during the course of the research will be kept strictly confidential. You will not be able to be identified in any ensuing reports or publications.

8. What will happen to the results of the research project? Following completion of the study, we aim to publish the results in a peer-reviewed journal and present the data at conferences and on social media. The anonymised data may be used by others for future research but no one will be able to identify you when this data is shared.

9. Local Data Protection Privacy Notice. The controller for this project will be University College London (UCL). The UCL Data Protection Officer provides oversight of UCL activities involving the processing of personal data task in the public interest, and can be contacted at data-protection@ucl.ac.uk. This 'local' privacy notice sets out the information that applies to this particular study. Further information on how UCL uses participant information can be found in our 'general' privacy notice: For participants in research studies, click here. The information that is required to be provided to participants under data protection legislation (GDPR and DPA 2018) is provided across both the 'local' and 'general' privacy notices. Your

personal data will be processed so long as it is required for the research project. If you are concerned about how your personal data is being processed, or if you would like to contact us about your rights, please contact UCL in the first instance at data-protection@ucl.ac.uk. UCL's Data Protection Officer is Alex Potts - data-protection@ucl.ac.uk. Thank you for reading this information sheet and for considering to take part in this research study. Your consent is important to us. Please can you click on the consent button below.

Q2 **Please read carefully:**

I have read the above information page and understand what the study involves and
· I understand that if I decide at any time that I no longer wish to take part in this project, I can withdraw immediately by not submitting my answers. But once I have submitted, I cannot withdraw.
· consent to the processing of my anonymised personal information for the purposes of this research study.
· understand that such information will be treated as strictly confidential and handled in accordance with the provisions of the Data Protection Act 1998.
· agree that the research project named above has been explained to me to my satisfaction and I agree to take part in this study.
· agree that my data, which is fully anonymised, can be shared with other researchers.

Am I eligible?
Are you female?
Are you aged 40 or over?·

If you answered yes to the questions above, and are able to read and speak English, you will be eligible to take part in this study.

Consent: I confirm that I am a female aged 40 or over. I have read and agree with the statements above

○ Consent (1)

○ I do not consent (2)

Q3 **There are three parts to the survey. We would like to ask you about yourself, your experience of the menopause and learning about the menopause and finally some basic information about your background. It is really important that you complete all three parts as incomplete surveys cannot be included in our analysis. You will know when your survey results are submitted as you will receive a copy of the menopause poster. Thank you.**

Q4 **Please state your country of residence**

- ○ UK (4)
- ○ Other (5) ____

JS

Q5 **Please state your age in years:**

____

Q6 **Please state your gender identity.**

- ○ Female (1)
- ○ Non-binary (2)
- ○ Other (4)
- ○ Prefer not to say (3)

Q7 **Please state your sexual orientation.**

- ◯ Heterosexual (1)
- ◯ Homosexual (2)
- ◯ Bisexual (3)
- ◯ Pansexual (4)
- ◯ Asexual (5)
- ◯ Prefer not to say (6)

Q8 **Please select the option that most reflects your current relationship status.**

- ◯ Single (1)
- ◯ In a relationship not cohabiting (2)
- ◯ In a relationship cohabiting (3)
- ◯ Married/civil partnership (4)
- ◯ Widowed (9)
- ◯ Prefer not to say (7)
- ◯ Other - in your own words (8) ____

Q9 **Do you have children?**

- ○ 1 (1)
- ○ 2 (2)
- ○ 3 (3)
- ○ 4 or more (4)
- ○ I do not have children (5)
- ○ Prefer not to say (6)

---

*

Q10 **Relating to your wellbeing, tick all that apply.**

- ☐ I regularly exercise (1)
- ☐ I sleep well (2)
- ☐ I eat a healthy diet most of the time/always (3)
- ☐ I am a moderate alcohol drinker or do not drink at all (4)
- ☐ I do not smoke (5)
- ☐ I have good mental health (6)
- ☐ None of the above (8)
- ☐ Prefer not to say (7)

Q11 **Are you currently using any methods below as a form of contraception (do not tick if using for other reasons apart from contraception, such as for heavy periods).**

○ Female condom (1)

○ Male condom (2)

○ Combined pill (3)

○ Progesterone only (mini) pill (4)

○ Contraceptive Implant (5)

○ Contraceptive Injection (6)

○ Contraceptive Patch (7)

○ Vaginal Ring (8)

○ Diaphragm (9)

○ Intrauterine system (10)

○ Intrauterine device (11)

○ Fertility awareness methods (body temperature ovulation sticks) with an app (12)

○ Fertility awareness methods (body temperature ovulation sticks) without an app (13)

○ Withdrawal method (14)

○ Male sterilisation (15)

○ Female sterilisation (16)

○ None of the above (19)

Q12 **Part 2: We would like to ask you about your learning of the perimenopause/menopause, your experience and your attitude.**

Q13 **Which of the following stages do you think best describes you?**

○ Not in the perimenopause/menopause (1)

○ Peri-menopausal - still having periods with some peri-menopausal symptoms (2)

○ Post menopausal - not had a period for 1 year or more (5)

○ I am not sure if I am peri-menopausal/menopausal (6)

○ I do not currently have periods so I am not sure whether I am peri-menopausal/menopausal (3)

Q14 **How do you feel about the peri-menopause/menopause?**

○ Looking forward to it (6)

○ Accepting of it (7)

○ Neutral - no strong view either way (8)

○ Dreading it (9)

○ Not sure (10)

Q15 **What are your thoughts about no longer having periods?**

- ○ Happy (1)
- ○ Neutral - no strong view either way (2)
- ○ Will miss not having a period (7)
- ○ Not thought about it (5)
- ○ Other - Please tell us more in your own words (4) ________________

Q16 **Before you went through the menopause, how did you feel about it?**

- ○ Was looking forward to it (1)
- ○ Accepting of it (9)
- ○ Neutral - no strong views either way (2)
- ○ Was dreading it (3)
- ○ Not sure (10)

Q17 **Now you have been through the menopause, how do you feel about it?**

- ◯ It was very difficult (1)
- ◯ It was difficult (2)
- ◯ Neutral - no strong views either way (13)
- ◯ It was fine (14)
- ◯ Not sure (19)

Q18 **What are your thoughts about no longer having periods?**

- ◯ Happy (1)
- ◯ Neutral - no strong view either way (2)
- ◯ I miss not having a period (7)
- ◯ Not thought about it (5)
- ◯ Other - Please tell us more in your own words (4)

____

Q19 **How do you feel now you are post menopausal?**

- ◯ Worse than I felt before the menopause (1)
- ◯ Neutral - no strong views either way (2)
- ◯ Better than I felt before the menopause (3)

Q20 **When do you think the menopause should be taught? Tick all that apply.**

- ☐ School (5)
- ☐ University (8)
- ☐ Doctor's surgery (9)
- ☐ Contraception clinic (10)
- ☐ Apps such as period trackers and fertility apps (11)
- ☐ Pregnancy (12)
- ☐ Other - please give details (7)

____

Q21 **How were you taught about the menopause at school?**

- ☐ Very detailed (1)
- ☐ Basic (2)
- ☐ Not at all (3)

Q22 **Before the age of 40, how informed did you feel about perimenopause/menopause?**

- Very informed (1)
- Some knowledge (2)
- Not informed at all (3)
- Not sure (4)

Q23 **What age were you when you started to think about the perimenopause/menopause?**

- Please put the age when you started thinking about the perimenopause/menopause (10) ____
- I cannot remember (11)
- I have not started to think about the perimenopause/menopause (12)

Q24 **Have you specifically looked for information of the menopause in any of these ways – tick all that apply**

- ☐ Official web sites such as the menopause society (14)
- ☐ Other web sites (1)
- ☐ YouTube (2)
- ☐ Podcasts (13)
- ☐ Social media (11)
- ☐ Magazines (3)
- ☐ Newspapers (4)
- ☐ Books (5)
- ☐ Documentaries (6)
- ☐ Films and TV programs (7)
- ☐ Friends (8)
- ☐ Health professionals (9)
- ☐ Scientific literature (10)
- ☐ Other - please give details (12)

____

Q25 **If you did look for information about the perimenopause or the menopause, at which stage did you look for it?**

- ○ Before I had symptoms (2)
- ○ As my symptoms started (1)
- ○ A long time after my symptoms started (5)
- ○ I have not looked for any information yet (4)

Q26 **Have you had, or are you having, any of the symptoms below since becoming peri-menopausal or menopausal – tick all that apply:**

- ☐ Hot flushes (12)
- ☐ Night sweats (1)
- ☐ Irregular periods (2)
- ☐ Heavy periods (32)
- ☐ Painful periods (33)
- ☐ Mood swings (7)
- ☐ Depression (27)
- ☐ Anxiety (21)
- ☐ Paranoia (23)
- ☐ Brain fog (30)
- ☐ Difficulty/poor concentration (46)
- ☐ Headaches/migraines (19)
- ☐ Poor memory (14)
- ☐ Tearful (36)
- ☐ Irritability (58)
- ☐ Low mood (31)
- ☐ Reduced confidence (54)
- ☐ Dizziness (15)

- [ ] Insomnia/problems sleeping (20)
- [ ] Fatigue (8)
- [ ] Lack of motivation (53)
- [ ] Low sex drive (45)
- [ ] Loss of sex drive (3)
- [ ] Vaginal dryness (4)
- [ ] Vaginal problems (5)
- [ ] Itching - ears, body, vagina, anywhere (6)
- [ ] Tingling in hands/arms/legs/feet (29)
- [ ] Clammy feeling (44)
- [ ] Heart palpitations (24)
- [ ] Weight gain (16)
- [ ] Bloating (41)
- [ ] Digestive issues (48)
- [ ] Osteoporosis (35)
- [ ] Aching joints (17)
- [ ] Aching muscles (18)
- [ ] Muscle tension (47)
- [ ] Restless leg syndrome (28)
- [ ] Burning tongue/roof of mouth (25)

- [ ] Gum problems (40)
- [ ] Bad Breath (51)
- [ ] Ears ringing (tinnitus) (52)
- [ ] Facial hair growth (49)
- [ ] Hair loss/thinning (59)
- [ ] Body odour (39)
- [ ] Incontinence (37)
- [ ] Urinary symptoms (55)
- [ ] Breast soreness (38)
- [ ] Weak nails (57)
- [ ] Brittle nails (42)
- [ ] Cold Flushes (43)
- [ ] Electric shock sensations (50)
- [ ] Increased allergies (56)
- [ ] I have not had any of these symptoms (26)
- [ ] Other - please state in your own words (34)

____

Q27 **Have you spoken with a health professional about the peri/menopause?**

- ○ Yes (1)
- ○ No (2)
- ○ Aiming to soon (3)

Q28 **Have you used any of these methods to alleviate perimenopausal/menopausal symptoms? Tick all that apply.**

- ☐ Nutrition changes including reduced alcohol/caffeine (1)
- ☐ Exercise (11)
- ☐ Hormone replacement therapy (HRT) (2)
- ☐ Bio identical hormones (6)
- ☐ Compounded hormones (7)
- ☐ Intrauterine device (Mirena coil) (8)
- ☐ Topical oestrogen (9)
- ☐ Cognitive behavioural therapy (12)
- ☐ Serotonin uptake inhibitors (13)
- ☐ Complementary therapies such as homeopathy, acupuncture, etc - please give details (5) ____
- ☐ Other - please give details (4) ____
- ☐ Not used any of these methods (14)

Q29 **In your own words, would you like to tell us anything about your views of the peri-menopause/menopause?**

____

____

____

____

____

Q30 **Part 3: Finally, we need to know a little about your background so we can compare different groups of people. It is really important that you complete this section, as otherwise we cannot use your answers. You will know when the survey is submitted as you will receive a copy of the menopause poster.**

Q31 **What is your highest educational qualification?**

- ○ Secondary School (1)
- ○ A Level/College-level (6)
- ○ University undergraduate (2)
- ○ University postgraduate (3)
- ○ Other (4) ____
- ○ Prefer not to say (5)

Q32 **What is/was your field of study/work/trade?**

____

Q33 **What is your religion or belief:**

○ No religion or belief (1)

○ Christian including Church of England, Catholic, Protestant and all other Christian denominations (2)

○ Hindu (3)

○ Jewish (4)

○ Muslim (5)

○ Sikh (6)

○ Buddhist (7)

○ Any other religion or belief - please give details (8) ________________

○ Prefer not to say (9)

Q34 **How do you identify yourself? Choose one or more.**

☐ White - English / Welsh / Scottish / Northern Irish / British (1)

☐ White - Irish (2)

☐ Any other White background (please specify) (3) ________________

☐ Black/Black British - African (4)

☐ Black/Black British - Caribbean (10)

☐ Any other Black/African/Caribbean background (please specify) (11) ________________

☐ Latino (16)

☐ Asian/Asian British - Indian (12)

- [ ] Asian/Asian British - Pakistani (13)
- [ ] Any other Asian background (please specify) (5)

____

- [ ] Arab (8)
- [ ] Mixed ethnic background (please specify) (7)

____

- [ ] Any other ethnic group, please describe (9)

____

- [ ] Prefer not to say (15)

Q35 **What is your disability status?** The Equality Act 2010 states a person has a disability if they have a physical or mental impairment that has a substantial and long-term adverse effect (likely to last 12 months or more) on their ability to perform normal day-to-day activities (e.g. eating, washing, walking and going shopping).

☐ No disability (1)

☐ Sensory impaired (2)

☐ Physical or mobility impaired (3)

☐ Specific learning difficulty or disability (e.g. dyslexia) (4)

☐ General learning disability (cognitive) (5)

☐ Long term illness or health condition (6)

☐ Autistic spectrum disorder (7)

☐ Other, please specify (8)

____

☐ Prefer not to say (9)

Q36

You will now be directed to the Menopause Poster produced by Pausitivity. Thank you for your time in completing our survey. We value and appreciate your participation. If you would like to discuss any of the issues that came up in this questionnaire, please know that there are sources of support available. In the first instance, you may wish to contact your doctor. Samaritans - https://www.samaritans.org/how-we-can-help/- An organisation dedicated to providing support when experiencing distress or worries.

____

____

____

____
